# Endometrial preservation during resection of type II and type III submucosal fibroids

**DOI:** 10.52054/FVVO.14.3.038

**Published:** 2022-09-30

**Authors:** G Vorona, E Saridogan

**Affiliations:** University College London Hospital, Women’s Health Division, London, United Kingdom; University College London, Elizabeth Garrett Anderson Institute for Women’s Health, London, United Kingdom; NIHR University College London Hospitals Biomedical Research Centre, London, United Kingdom

**Keywords:** submucosal myoma, transcervical resection, endometrium, infertility

## Abstract

**Background and objectives:**

Hysteroscopic myomectomy is considered the gold-standard treatment of submucosal fibroids. However, it is associated with disruption of the endometrium which may lead to complications such as intrauterine adhesions and loss of functional endometrium. In this video article we describe a technique to resect Type III and Type II fibroids whilst minimising the loss of overlying endometrium.

**Materials and Methods:**

We present two patients with type II/III submucosal fibroids with minimal or no intracavity component. The resection technique we demonstrate comprises either making an endometrial incision or making a small opening in the overlying endometrium to expose the fibroid pseudocapsule. Subsequent steps of resection are then performed through this small opening. Thus, complete resection is achieved without further resection of the endometrium.

**Main outcome measures:**

Evidence of endometrial healing and absence of intrauterine synechiae on follow up outpatient hysteroscopy or ultrasound scan.

**Results:**

Full resection was achieved in both patients with no or minimal loss of overlying endometrium. A follow up outpatient hysteroscopy was performed 8 weeks later in the first patient, demonstrating completely healed uterine cavity. She had a successful conception and delivery following IVF treatment for male factor infertility. The second patient is currently in the process of IVF treatment.

**Conclusions:**

Our technique enables endometrial preservation and potentially better reproductive outcomes following resection of type II and type III submucosal fibroids. Larger scale studies are required to elucidate long term outcomes on bigger patient population.

## Learning objective

We propose and demonstrate a technique to preserve the endometrium during resection of type III and type II submucosal fibroids with minimal intracavity component. This method involves minimising or avoiding loss of endometrium during the process. This can be achieved either by making an incision over the endometrium or resecting a small surface area over the fibroid to expose the fibroid pseudocapsule. The exposed area needs to be wide enough for the resectoscope to enter the pseudo capsule so that the fibroid can be removed through this small opening. Once the fibroid is exposed, the loop of the resectoscope is used to resect the fibroid whilst remaining within the pseudocapsule until complete resection is achieved.

## Introduction

Hysteroscopic myomectomy is considered the gold-standard treatment option of the submucosal fibroid. Transcervical resection of fibroids (TCRF) is frequently used for the treatment of women with heavy menstrual periods or infertility. Amongst the merits of TCRF are the minimally invasive approach, same-day discharge and quick recovery. However, the procedure is associated with disruption of the endometrium, and subsequent complications such as intrauterine adhesions and loss of functional endometrium. Whilst intrauterine adhesions following TCRF is a well-recognised problem, loss of functional endometrium is not widely appreciated and there is no significant data in the literature (Garcia-Velasco et al., 2016). Loss of functional endometrium usually represents itself as thin or hyperechogenic endometrium in natural or assisted reproduction (ART) cycles.

In experienced hands, the risk of significant injury to the endometrium is minimal during resection of type 0 submucosal fibroids and low for type I fibroids. However, resection of type II and type III fibroids is more challenging as more than 50% of the fibroid is intramural, and quite often, a large section of the overlying endometrium is resected to access the fibroid. This can result in a lack of proper endometrial development postoperatively.

Literature on techniques that preserve the functional endometrium during TCRF, especially for type II (≥50% intramural) fibroids, is scarce (Di Spiezio Sardo et al., 2008). In this video article we describe a technique to resect Type II and Type III fibroids whilst minimising loss of overlying endometrium.

## Patients and methods

In this video article we describe two patients with type II and III submucosal fibroids with minimal intracavity component. The first patient was a 35-year-old woman with a history of primary infertility and heavy menstrual bleeding. She was due to undergo IVF treatment for male infertility and was diagnosed with a 3 cm posterior wall fibroid which was found to be encroaching the endometrium. Initial hysteroscopic examination showed the cavity looked mostly normal but it was possible to locate the fibroid in the lower part of the posterior wall, giving the appearance of a type III fibroid. A small opening in the overlying endometrium was made by resecting a limited area to expose the fibroid pseudocapsule. Subsequent steps of resection were taken through this small opening without resecting the endometrium further. The fibroid was then resected completely whilst remaining in the fibroid pseudocapsule.

The second patient was a 41-year-old woman who had a 1.5 cm type II fibroid located in the left lateral wall of the uterine cavity. She was due to undergo IVF treatment and her ultrasound scan showed that 80-90% of the fibroid was intra-myometrial. At initial hysteroscopy the location of the fibroid was not obvious, hence the distension fluid was turned off to see the protrusion of the fibroid. Once the fibroid was located, an incision was made in the endometrium just over the fibroid without resecting any endometrium. This exposed the fibroid and the pseudocapsule around it. Small chips of fibroids were resected through this opening in combination with mechanical mobilisation of the fibroid to dislodge the fibroid and make it easier to resect.

## Results

Complete resection was achieved in both patients with no or minimal loss of overlying endometrium. A follow up outpatient hysteroscopy was performed 8 weeks later in the first patient, and this showed that the uterine cavity was completely healed, without any obvious sign of previous fibroid resection. She since conceived following IVF treatment and had a successful delivery. The second patient is currently in the process of IVF treatment, her ultrasound examinations showed normal endometrial development.

## Discussion

Submucosal fibroids are known to reduce implantation and clinical pregnancy rates in natural and ART cycles, and their removal improves outcomes (Pritts et al., 2009; Saridogan and Saridogan, 2019). Limited data related to Type III fibroids also suggest their presence reduces chance of implantation, clinical pregnancy, and live birth rates in ART cycles (Yan et al., 2018). Hence hysteroscopic resection of these fibroids is quite often performed.

The healing process following fibroid resection has not been systematically studied. Limited published data and clinical experience from repeat hysteroscopies following initial resection show that the crater (or fibroid bed) fills and is usually covered with endometrium in the following 2-3 months after surgery (Yang et al., 2013). It can be assumed that smaller endometrial defects are easier and quicker to heal compared to larger defects. It is also likely that larger defects may be covered with suboptimal endometrium, and this can affect reproductive function postoperatively. The published literature concentrates on intrauterine adhesion development following hysteroscopic myomectomy and limited data suggest that adhesion development is seen in between 7.5% and 31.3-45.5% of women undergoing this procedure (31.% after resection of single myoma and 45.5% after multiple myomas) (Taskin et al., 2000; Touboul et al., 2009).

To our knowledge there are no data on endometrial histology, thickness or echogenicity following fibroid resection. Anecdotally, thin endometrium is more likely to be seen in this patient group. Theoretically, modifying the surgical technique to minimise or avoid overlying endometrium resection would be expected to avoid this detrimental impact on endometrium. Admittedly, our technique makes the resection process more difficult, and it probably takes longer compared to the standard technique in which the overlying endometrium is completely resected to expose the fibroid. Furthermore, the technique requires advanced hysteroscopic surgery skills and should probably be left to those with extensive experience. Further studies are required to increase our understanding of healing of uterine cavity, particularly the endometrium, following hysteroscopic myomectomy.

## Conclusions

We believe this approach offers better endometrial preservation and possibly better reproductive function following resection of type II and type III submucosal fibroids.

## Video scan (read QR)

https://vimeo.com/684240723/e6eae2888a

**Figure:**
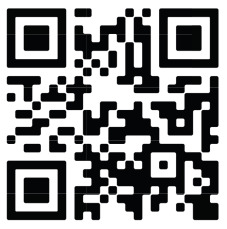
QR code resolving to https://vimeo.com/684240723/e6eae2888a
